# AHAPS-functionalized silica nanoparticles do not modulate allergic contact dermatitis in mice

**DOI:** 10.1186/1556-276X-9-524

**Published:** 2014-09-24

**Authors:** Anja Ostrowski, Daniel Nordmeyer, Lars Mundhenk, Joachim W Fluhr, Jürgen Lademann, Christina Graf, Eckart Rühl, Achim D Gruber

**Affiliations:** 1 Institute of Veterinary Pathology, Freie Universität Berlin, Robert-von-Ostertag-Str. 15, 14163 Berlin, Germany; 2 Institute of Chemistry and Biochemistry - Physical and Theoretical Chemistry, Freie Universität Berlin, Takustr. 3, 14195 Berlin, Germany; 3 Department of Dermatology, Venerology and Allergology, Charité - Universitätsmedizin Berlin, Charitéplatz 1, 10117 Berlin, Germany

**Keywords:** Allergic contact dermatitis, Oxazolone, Toxicopathology, Mouse model, Silica nanoparticles

## Abstract

Allergic contact dermatitis (ACD) is a common skin disease in people and may become a potential site of exposure to nanoparticles (NP). Silica nanoparticles (SiO_2_-NP) possess a promising potential for various medical and non-medical applications, including normal and diseased skin as target organs. However, it has been shown that negatively charged SiO_2_-NP may act as proinflammatory adjuvant in allergic diseases. The effect of topical SiO_2_-NP exposure on preexisting ACD has not been studied to date although this reflects a common *in vivo* situation. Of particular interest are the potential effects of positively charged *N*-(6-aminohexyl)-aminopropyltrimethoxysilane (AHAPS)-functionalized SiO_2_-NP which are promising candidates for delivery systems, including gene delivery into the skin. Here, the effects of such AHAPS-functionalized SiO_2_-NP (55 ± 6 nm in diameter) were studied in an oxazolone-induced ACD model in SKH1 mice and compared to ACD mice treated with vehicle only. The clinical course of the disease was assessed by monitoring of the transepidermal water loss (TEWL) and the erythema. In histologic and morphometric analyses, the distribution of particles, the degree of inflammation, epidermal thickness, and the inflammatory infiltrate were characterized and quantified by standard and special histological stains as well as immunohistochemistry for CD3+ lymphocytes. To assess possible systemic effects, serum immunoglobulin E (IgE) was determined by enzyme-linked immunosorbent assay. Following administration of AHAPS-SiO_2_-NP for five consecutive days, no effects were observed in all clinical, histologic, morphometric, and molecular parameters investigated. In conclusion, positively charged AHAPS-SiO_2_-NP seem not to affect the course of ACD during exposure for 5 days.

## Background

Silica nanoparticles (SiO_2_-NP) are among the most promising inorganic nanoparticles (NP) for biomedical applications, such as drug and gene delivery systems, including vaccination [1,2]. In addition, they are used in everyday products like cosmetics [3].

Several studies have shown pro-inflammatory adjuvant effects of SiO_2_-NP during ovalbumin or mite antigen-induced allergic dermatitis and allergic airway disease when NP and antigen were administered simultaneously as immunogenic challenge [4-8]. However, no study has addressed possible effects of intended or unintended NP exposure in an already existing allergic contact dermatitis (ACD) which affects approximately 20% of European and North American people [9].

To date, the *in vivo* adjuvant effects of SiO_2_-NP in allergic disease models have only been studied for negatively charged SiO_2_-NP, including unfunctionalized and polyethylene glycol (PEG)-functionalized SiO_2_-NP [4-8]. However, zeta potentials indicative of biologically relevant surface charges have not always been reported in these studies. The surface charge of NP and thus several aspects of their performance in bioenvironments can largely be modified by different functionalizations [10]. For example, surface functionalization of SiO_2_-NP may drastically reduce their *in vitro* and *in vivo* cytotoxicity compared to unfunctionalized SiO_2_-NP [10,11]. Moreover, positively charged *N*-(6-aminohexyl)-aminopropyltrimethoxysilane (AHAPS)-functionalization results in increased colloidal stability compared to unfunctionalized SiO_2_-NP and markedly reduces its tendency towards aggregation [12]. Accordingly, it appears reasonable to assume that such surface functionalization may also influence NP behavior in diseased tissues, including ACD. Furthermore, AHAPS-functionalization with positive surface charge also allows for additional applications, including DNA binding for gene delivery or vaccination approaches [13]. However, such particles have not been tested in an ACD environment to date.

Consequently, in the present study, we investigated the effects of positively charged AHAPS-SiO_2_-NP in a murine model of acute oxazolone-induced ACD [14].

## Methods

### Particle synthesis and characterization

**Figure 1:**
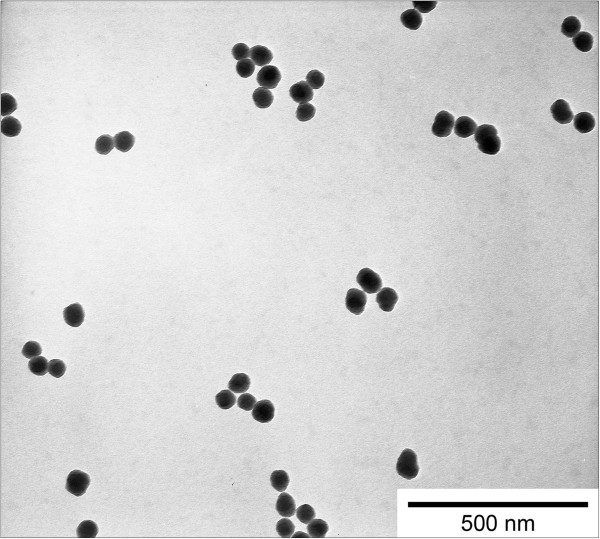
**Dispersion characteristics of AHAPS-SiO**_**2 **_**nanoparticles.** Transmission electron microscopy revealed well-dispersed FITC-labeled AHAPS-SiO_2_-NP with a diameter of 55 ± 6 nm.

Spherical SiO_2_-NP with a diameter of 55 ± 6 nm were grown in a seeded growth process around a fluorescein-5-isothiocyanate (FITC)-labeled SiO_2_-NP core as reported previously [15,16]. Surface functionalization with AHAPS bearing one primary and one secondary amino group per molecule resulted in NP of high colloidal stability [12] and changed the zeta potential of the SiO_2_-NP from highly negative −45 ± 4 mV to highly positive values +37 ± 2 mV. Well-dispersed AHAPS-SiO_2_-NP were transferred to ultra-pure water (AlleMan Pharma, Pfullingen, Germany) and remained colloidally stable as indicated by transmission electron microscopy (Figure 1) as well as dynamic light scattering (DLS) measurements of hydrodynamic diameter and zeta potential as previously shown [17].

### Animals and study protocol

The study protocol was approved by the State Office of Health and Social Affairs, Berlin (LAGeSo G 0209/11) and adhered to national guidelines. ACD was induced in 6- to 8-week-old, male mice of the outbred SKH1 strain (*n* = 5; Charles River, Germany) using topical application of oxazolone (Ox; Sigma-Aldrich, Steinheim, Germany) in acetone (Biesterfeld Chemiedistribution, Hamburg, Germany). The animals were sensitized with 1.5% Ox in acetone on the right flank. One week later, a first challenge with 0.5% Ox in acetone was applied to the right flank, followed by a second challenge with 0.5% Ox in acetone 2 days later. Each animal was treated on five consecutive days with 50 μL AHAPS-SiO_2_-NP (*c* = 5 g/L) or ultra-pure water, respectively, per day starting 12 h following the first challenge as previously described in [17]. A solvent group (*n* = 3) was treated with acetone only to control the effects of Ox. Prior to each treatment, the transepidermal water loss (TEWL) was measured to assess the skin barrier disruption with a Tewameter TM 300 (Courage + Khazaka, Cologne, Germany) as described in [18]. Erythema was determined macroscopically and a photograph was taken daily with a digital camera (NEX-3, Sony, Tokyo, Japan). Twenty-four hours after the last treatment, animals were euthanized and tissues were sampled as described in [17].

### IgE measurements

Blood samples were collected by cardiac puncture immediately following euthanasia and serum immunoglobulin E (IgE) concentrations determined with a mouse IgE enzyme-linked immunosorbent assay (ELISA) quantification kit (Bethyl Laboratories, Montgomery, TX, USA) according to the manufacturer's protocol [19].

### Particle localization and histologic and morphometric analyses

Fluorescent AHAPS-SiO_2_-NP were localized in 4′,6-diamidino-2-phenylindole (DAPI; Carl Roth, Karlsruhe, Germany) stained section as described in [17]. Furthermore, serial 5-μm sections of formalin-fixed, paraffin-embedded skin were stained following routine protocols with hematoxylin and eosin (HE) and toluidine blue or Congo red for visualization of mast cells or eosinophils, respectively. T lymphocytes were immunohistochemically identified in lesional skin using antibodies to the surface marker CD3 (rabbit polyclonal anti-human CD3, dilution 1:1,500, Dako, Glostrup, Denmark) following heat-induced antigen retrieval with citrate buffer (pH 6.0) as reported in [20].

Samples were examined microscopically using a BX41 microscope (Olympus, Shinjuku-ku, Japan) equipped with a digital camera (Color-View II, SIS, Münster, Germany), a fluorescence burner (U-RLF-T, Olympus, Japan), and a digital image analysis software (AnalySIS docu, version 5.0, SIS, Münster, Germany). Tissues were evaluated histopathologically and cells were quantified in an observer-blinded manner as described below.

Histologic analysis of lesional skin included the evaluation of hyperkeratosis, parakeratosis, intraepidermal exocytosis, and dermal infiltrate. Epidermal thickness was measured in six randomly selected lesional areas from the basement membrane to the base of the stratum corneum as described in [21]. Mast cells and eosinophils were counted in 20 randomly selected high-power fields (hpf) at × 400 magnification. T lymphocytes were counted in 10 hpf per epidermis and dermis, respectively.

### Statistical analyses

Data are presented as mean ± standard error of the mean (SEM). Statistical analyses were performed with SPSS software (IBM, Armonk, NY, USA) using one-way ANOVA for normally distributed parameters (IgE, epidermal thickness, and CD3+ cells) or Kruskal-Wallis test for not normally distributed parameters (mast cells and eosinophils). For all tests, significance was considered for *p* < 0.05 with the following designation: *p < 0.05 and ***p < 0.001.

## Results

**Figure 2:**
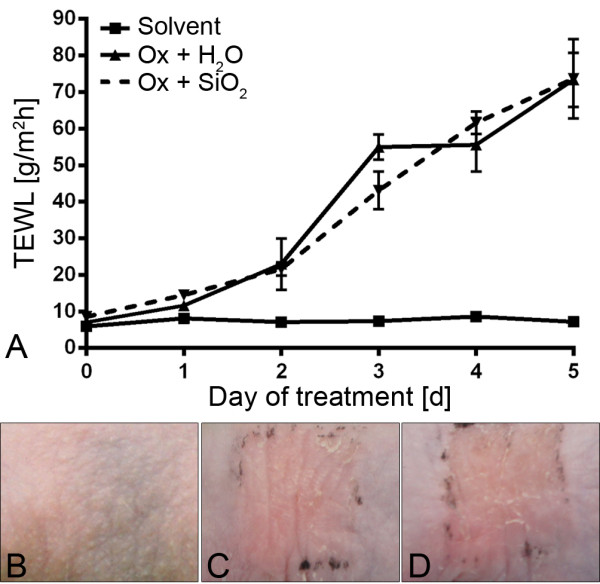
**Effects of AHAPS-SiO**_**2**_**-NP treatments on barrier function and erythema.** Transepidermal water loss (TEWL) increased in oxazolone (Ox)-treated mice without significant differences between the AHAPS-SiO_2_-NP-treated versus ultra-pure water-treated groups. Solvent-only-treated mice failed to develop elevated TEWL values above the physiological threshold **(A)**. Erythema was absent from the skin of the acetone solvent group **(B)** but present to similar degrees in the vehicle control group **(C)** and AHAPS-SiO_2_-NP-treated group **(D)**. Data are presented as mean ± SEM; Ox-treated groups: *n* = 5; solvent-treated group: *n* = 3.

**Figure 3:**
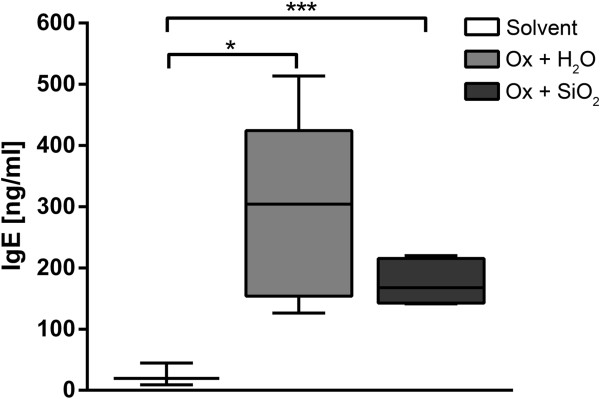
**Serum IgE response following topical oxazolone treatment.** Serum IgE levels were significantly different between the acetone solvent control and oxazolone (Ox)-treated groups but without significant differences between the vehicle and AHAPS-SiO_2_-NP treatments. Data are presented as a box plot with whiskers displaying minimum to maximum values; Ox-treated groups: *n* = 5; solvent-treated group: *n* = 3; *p < 0.05, ***p < 0.001.

Clinically, the Ox treatment resulted in a marked increase in TEWL values, independently of subsequent treatment with AHAPS-SiO_2_-NP or vehicle alone (Figure 2A). The highest values were recorded on treatment day 5 without significant differences between the two treatment groups (AHAPS-SiO_2_-NP versus ultra-pure water). Treatment of the skin with acetone alone did not increase TEWL values, and values remained within physiological ranges below 10 g/m^2^h [18]. Ox-treated skin but not the skin in the acetone solvent group developed a marked erythema beginning 12 h after the first Ox challenge and lasting for the whole treatment period (Figure 2B,C,D). Consistently, significant higher final serum IgE concentrations were recorded in Ox-treated mice compared to mice from the control group (Figure 3). There were no statistically significant differences between the AHAPS-SiO_2_-NP (176.69 ± 36.83 ng/mL) and vehicle (292.14 ± 150.59 ng/mL) treatments.

Using fluorescence microscopy of skin sections, the green fluorescent AHAPS-SiO_2_-NP were localized only in the superficial layers of the stratum corneum in all particle-treated mice as shown earlier [17]. No fluorescent signal was detected in the stratum corneum of ultra-pure water-treated animals.

**Figure 4:**
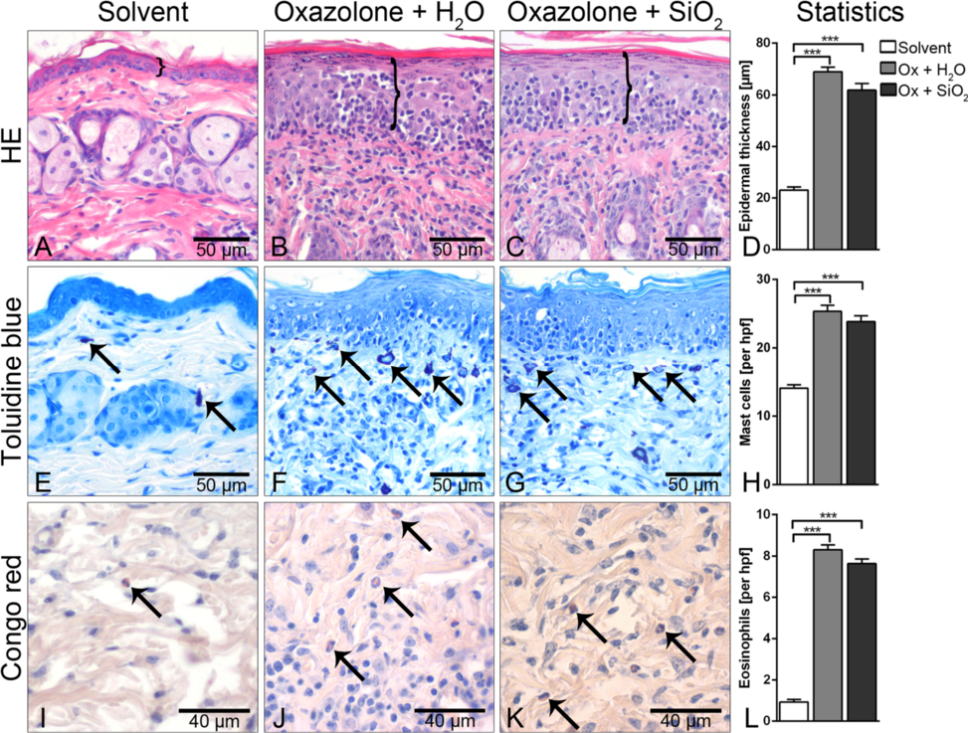
**Histologic and morphometric analyses of lesional skin.** Hematoxylin and eosin (HE) staining and measurement of epidermal thickness indicated by brackets (top panel) failed to reveal inflammation-associated epidermal thickening in solvent controls **(A)** but showed marked thickening of the epidermis and intraepidermal immune cell exocytosis in oxazolone (Ox)-treated mice without differences between the vehicle control and AHAPS-SiO_2_-NP treatments **(B-D)**. Increased infiltrations with mast cells (toluidine blue, central horizontal panel: **E-H**) and eosinophils (Congo red, bottom panel: **I-L**, arrows) were very similar between the two Ox-treated groups. Only a few mast cells and eosinophils were observed in acetone solvent controls **(E, I)**. Images represent typical samples. Quantification included all samples in panels D, H, and L. Data are presented as mean ± SEM; Ox-treated groups: *n* = 5; solvent-treated group: *n* = 3; ***p < 0.001.

**Figure 5:**
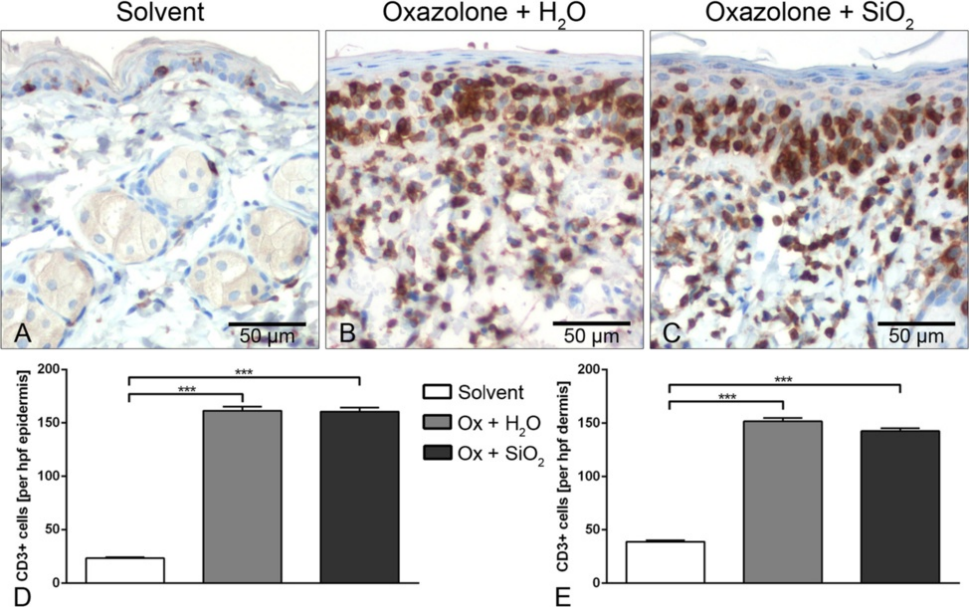
**Quantification of T lymphocytes.** Cellular infiltrates of the skin in acute contact dermatitis were mainly attributed to T lymphocytes as identified immunohistochemically by the detection of CD3 antigen and visualized with the brown chromogen diaminobenzidine. Nuclei were counterstained with hemalaun. Significant differences were observed only between the acetone solvent control **(A)** and oxazolone (Ox)-treated groups but not between the vehicle control **(B)** and AHAPS-SiO_2_-NP-treated groups **(C)**. The same quantitative effects were seen for T lymphocytic infiltration into the epidermis **(D)** and dermis **(E)**. Data are presented as mean ± SEM; Ox-treated groups: *n* = 5; solvent-treated group: *n* = 3; ***p < 0.001.

Histologically, the lesional skin of ACD mice showed a mild hyperkeratosis and a severe intraepithelial exocytosis as well as dermal infiltrates. Parakeratosis was minimal in Ox-treated animals. Mice treated with acetone solvent only did not show any histopathological changes. No differences in the histologic parameters were seen between both treatment groups of ACD (Figure 4A,B,C). Morphometrically, the epidermis in Ox-induced ACD was significantly thicker compared to that from mice of the solvent group. In contrast, no significant differences were recorded between epidermal thicknesses in AHAPS-SiO_2_-NP-treated versus vehicle-treated ACD mice (Figure 4A,B,C,D). Similar results were obtained for the numbers of mast cells and eosinophils (Figure 4E,F,G,H,I,J,K,L) as well as for T lymphocytes infiltrating the epidermis and dermis (Figure 5). The data of each group for all morphometric, histologic, and immunohistochemical parameters are summarized in Table 1.

**Table 1:** Morphometry and quantification of data from lesional skin

	**Solvent**	**Oxazolone + H**_**2**_**O**	**Oxazolone + AHAPS-SiO**_**2**_**-NP**
Epidermal thickness [μm]	23.04 ± 1.21	68.95 ± 1.7	61.84 ± 2.58
Mast cells^a^	14.08 ± 0.51	25.34 ± 0.9	23.84 ± 0.86
Eosinophils^a^	0.93 ± 0.12	8.30 ± 0.24	7.63 ± 0.23
T lymphocytes^a^ in the epidermis	23.33 ± 1.05	161.36 ± 3.87	160.52 ± 3.65
T lymphocytes^a^ in the dermis	38.63 ± 1.33	151.52 ± 3.19	142.54 ± 2.71

^a^Number of cells per high-power field (hpf), counted in at least 10 hpf. Epidermal thickness measurements and quantification of mast cells, eosinophils, and CD3+ T lymphocytes in the epidermis and dermis of lesional skin expressed as mean ± SEM.

## Discussion

In contrast to previous studies according to which skin barrier disruptions result in increased penetration of irritants with aggravation of inflammatory disease [22], we could not detect any effects of AHAPS-SiO_2_-NP on the quality or degree of inflammation. Specifically, our results are at variance with previous studies using negatively charged SiO_2_-NP that had aggravated allergic reactions in the skin and airways including the same dose as used here [4-8]. Among possible explanations of this discrepancy is the fact that AHAPS-SiO_2_-NP do not penetrate beyond the stratum corneum, the outermost layer of the skin, in normal and irritated skin, including the mice used here. The distribution and levels of penetration of AHAPS-SiO_2_-NP on the skin of ACD mice have been studied in detail in a previous report of our group [17]. In that study, no pathological changes were recorded in healthy mice following exposure to AHAPS-SiO_2_-NP [17]. Furthermore, NP had been injected intradermally in the majority of previous studies [5,6]. Consequently, in these studies, the SiO_2_-NP reach viable epidermal layers and may directly interact with keratinocytes and immune cells. Thus, an injection poorly models the *in vivo* situation in human and murine ACD where NP are topically applied to the skin and have to overcome the relatively impermeable stratum corneum [23] especially when the thickness is increased due to hyperkeratosis. External, topical application of NP was reported only in a single study so far [7] in which a mild aggravation of skin lesions was observed. However, in that study, the SiO_2_-NP were applied simultaneously with mite antigen over a 4-week period with three applications per week. So far, the effects of topical SiO_2_-NP exposure to an already existing ACD have not been studied although ACD is a common skin disorder in Western European and North American people with a prevalence of about 20% [9]. As reported by several studies, even higher values for IgE, mast cells, eosinophils, and CD3-positive cells could have been expected in inflamed mouse skin. It therefore seems likely that NP-induced aggravation of the ACD would still have been detectable in the background of our Ox-induced inflammation [5,24-26]. Furthermore, it has been shown that surface functionalization improves the biocompatibility of SiO_2_-NP [10,11]. Therefore, both the failure of penetrating beyond the stratum corneum and the improved biocompatibility due to functionalization may have prevented the aggravation of barrier defects and inflammatory response in our study. However, we cannot exclude the possibility that effects would have occurred if significantly higher doses would have been used. Still, the dose used here appears to reflect a realistic condition and offers optimal comparability with the previous studies on unfunctionalized and functionalized SiO_2_-NP [5,8,17].

In addition, the specific model used here for the induction of ACD may have had an effect on the results. Ox-induced dermatitis cannot be strictly classified as either T helper (Th)1- or Th2-dominated response [27]. In contrast, ovalbumin and mite antigens result in specifically Th2-driven allergic dermatitis. However, the specific roles of the immune mechanisms involved, both in terms of induction of the hypersensitivity reaction and the exacerbation of allergic disease by certain NP other than AHAPS-SiO_2_-NP, need to be studied in the future.

## Conclusions

Taken together, our data show that AHAPS-SiO_2_-NP exposure to diseased skin in an ACD model does not affect the course and outcome of the disease over 5 days. It thus seems that a short-time exposure of AHAPS-SiO_2_-NP to mouse skin is without any pathological consequences, at least as far as can be judged with the techniques employed here. The reasons why the AHAPS-functionalized NP do not modulate barrier disruption or inflammatory responses as seen in other allergic disease models and whether the observations hold true in a long-term exposure model should be addressed in the future.

## Abbreviations

ACD: Allergic contact dermatitis; AHAPS: *N*-(6-aminohexyl)-aminopropyltrimethoxysilane; DAPI: 4′,6-diamidino-2-phenylindole; ELISA: Enzyme-linked immunosorbent assay; FITC: Fluorescein-5-isothiocyanate; HE: Hematoxylin and eosin; IgE: Immunoglobulin E; NP: Nanoparticles; Ox: Oxazolone; PEG: Polyethylene glycol; SiO_2_-NP: Silica nanoparticles; TEWL: Transepidermal water loss; Th: T helper cells.

## Competing interests

The authors declare that they have no competing interests.

## Authors’ contributions

AO carried out the animal experiments, participated in the design of the study; conducted the histologic, morphometric, and immunohistochemical analyses; performed the ELISA and statistical analyses of all data; and drafted the manuscript. DN, CG, and ER synthesized, characterized, and provided AHAPS-SiO_2_-NP and helped to draft the manuscript. LM helped with the necropsy of animals, participated in the design of the study and data analyses, and helped to draft the manuscript. JWF and JL gave conceptual advice and participated in the design of the study. ADG supervised the project, participated in the design of the study, and helped to draft the manuscript. All authors discussed the results and commented on the manuscript. All authors read and approved the final manuscript.
